# Modelling, docking and simulation analysis of Bisphenol A interaction with laccase from Trichoderma

**DOI:** 10.6026/97320630016323

**Published:** 2020-04-30

**Authors:** Sankar Ganesh Ramakrishnan, Muthusaravanan Sivaramakrishnan, Dhrisya Chenthamara, Ram Kothandan, Swaminathan Krishnaswami, Sadhasivam Subramaniam

**Affiliations:** 1 Department of Microbial Biotechnology, Bharathiar University, Coimbatore, India; 2 Department of Extension and Career Guidance, Bharathiar University, Coimbatore, India; 3 Department of Biotechnology, Kumaraguru college of Technology, Coimbatore, India

**Keywords:** Laccase, Bisphenol-A, molecular simulation, docking

## Abstract

Fungal laccases are widely known for the degradation of recalcitrant xenobiotic compounds. Hence, it is of interest to study the interaction between laccase from Trichoderma laccase and Endocrine-Disrupting Chemical (EDC) named Bisphenol A. The molecular docking analysis of laccase from Trichoderma laccase with 23 xenobiotics and bisphenol A was completed. We show Bisphenol having optimal binding features (Glide score of -5.44 and the Glide energy -37.65 kcal/mol) with laccase from Trichoderma laccase.

## Background:

For the past few decades, endocrine disrupting compounds (EDCs) or xenoestrogens have been widely introduced into the environment mainly through anthropogenic activities. Among these compounds, Bisphenol A (BPA) (2, 2-bis (4-hydroxyphenyl) propane) is an ubiquitous endocrine disrupting chemical present as a primary monomer in most of the day to day consumables [1]. Bisphenol A is a carbon-based synthetic compound containing two 4-hydroxyphenyl rings [2]. It is useful in the production of polycarbonate plastic and epoxy resins, as well as a polymerization inhibitor in polyvinyl chloride and as a color developer in thermal papers. The presence of low doses (sub nanogram) of BPA is associated with health problems such as cancer, cardiovascular disease and diabetes [3]. BPA may cause breakage of some double-stranded DNAs in humans [4]. The accumulation of BPA in the body can cause metabolic disorders such as low sex-specific neurodevelopment and hindrance of cellular pathway [5].

White-rot fungi and their ligninolytic enzymes such as laccases, lignin peroxidases (LiPs) or manganese-dependent peroxidases (MnPs) are capable of degrading aromatic compounds such as BPA [6]. Manganese peroxidase (MnP), horseradish peroxidase and laccase are effective in removing the estrogenic activities of bisphenol A [7]. Among these laccases is the most frequently studied enzyme as it uses molecular oxygen as the final electron acceptor, which is usually available in the environment [8, 9]. The importance of laccases as oxidative biocatalysts relies on the fact that, their reactions do not require cofactors such as H_2_O_2_ and hence they are regarded as a 'Green tool' [10]. Laccase (benzenediol: oxygen oxidoreductase; EC 1.10.3.2) belongs to the multicopper oxidases that facilitate the catalysis of a vast range of phenolic aromatic amines thus reducing their toxicity. Laccases mostly appear in the forms of monomers and dimers [11, 12]. Laccases oxidize aromatic phenols and amines such as phenols, polyphenols, anilines, aryl diamines, hydroxindols, benzenethiols and some inorganic ions such as [Mo(CN)_8_]^4^-, [Fe(CN)_6_]^4^-, [Os(CN)_6_]^4^- and [W(CN)_8_]^4^ [13]. Due to their relatively relaxed substrate specificity (oxidize higher redox potential compounds) and thermostability (optimal temperature as high as 80°C) they are of great interest for industrial applications including pulp bleaching, food processing, textile treatments, and bioelectrochemistry [14,15].

Enzymatic degradation of pollutant is receiving more attention because of its specificity, ease of control and environmental friendly process. Hence the molecular simulation study will pave the way to gain knowledge about the interaction/binding of ascomycete laccase with bisphenol A. The molecular docking and molecular dynamics (MD) simulations have been considered as the most established techniques for the study of the protein ligand interaction at the molecular level [16]. Molecular docking studies uses the principle of lock and key theory proposed by Fischer and is based on the ligand-receptor binding mechanism. It uses algorithms to predict the binding or interaction of ligand and receptor and provide a theoretical basis as well as reliability of degradation [17]. MD simulations have been devised as a useful method to obtain the structural and dynamic information on basidiomycete (Pleurotus ostreatus) laccase [18] and bacterial CotA laccase (Bacillus subtilis) [19]. The binding mode and interaction of bisphenol A and basidiomycete Trametes versicolor laccase were reported by docking studies [17]. A highly thermostable laccases was reported in Trichoderma asperellum for enhanced biohydrogen production [20]. Thermostability is one of the important characteristics of an enzyme and the thermostable laccases have shown catalytic robustness that favors its applicability for environmental remediation. Hence, it is of interest to study the interaction between laccase from Trichoderma laccase and endocrine-disrupting chemical (EDC) named bisphenol A.

## Materials and Methods:

All the computational work has been carried out on Ubuntu Linux 16.04 platform in HP-ProLiant ML-350 G6 workstation on Intel Xeon quad core of 2.00 GHz processor.

### Biological Data:

Three-dimensional (3D) structures of the protein and ligand are necessary for molecular docking and molecular dynamics simulation studies [21]. Since the 3D structure of Trichoderma laccase is yet to resolve, the structure of the laccase was modelled using the available amino acid sequence from UniProtKB [22] with the sequence ID of C5H3G0. The 3D structure of the compounds studied for binding with laccase was taken from PubChem [23] repository in SDF format (Supplementary Material).

### Molecular modelling of laccase enzyme:

Ab initio molecular modelling

The 3D structure of laccase was modeled using I-TASSER (Iterative Threading ASSEmbly Refinement) standalone tool [24]. I-TASSER tool was used for Ab initio modelling of protein structure. I-TASSER comprises different modules for building a protein structure which are briefly described as follows: i) LOMETS-to retrieve the template proteins of similar folds from the local PDB library, ii) SPICKER-to cluster the template fragments, iii) TM-align – for structural reassembly, iv) REMO- for H-bond optimization and building the final structure. In total 5 models were generated from the I-TASSER tool and the best model was taken for further studies. The modelled laccase structure was evaluated using Ramachandran plot generated by RAMPAGE server [25]. Ramachandran plot was used to assess the quality of the modeled protein structure by looking for steric clashes or geometrical errors on dihedral angles of protein atoms. The overall quality of the modeled structure was analyzed through Z-score by utilizing ProSA server [26] (https://prosa.services.came.sbg.ac.at/prosa.php).

### Molecular docking studies:

#### Protein structure preparation:

The protein (laccase) structure was prepared using protein preparation wizard present in the Maestro 11.7 version of Schrodinger suite [27]. The protein preparation is composed of fixing the structure and geometric errors, by optimizing the hydrogen bonds to keep all the atoms in the proper position by applying restrained energy minimization (impref). OPLS_2005 [28] force field was employed for energy minimization.

#### Ligand preparation:

Ligands were prepared using LigPrep module available in Maestro 11.7 version of Schrodinger suite [29]. Default parameters such as ionization state of pH 7.0, including tautomeric and stereo chemical variations were employed. During ligand preparation, chiralities of the ligands were corrected and the necessary hydrogen atoms were added to each and every ligand structure.

#### Active site identification:

The active site of the protein was identified by the SiteMap tool available in the Maestro 11.7 version of Schrodinger suite [30]. The site identified by the SiteMap with top score and larger volume is considered as an active site and used for further docking studies.

#### Receptor grid generation:

The grid generation was carried out using a grid generation panel available on the Glide module of Schrodinger suite [31]. The grid box was generated on the center of the active site of the protein. The default grid size of 30 Å x 30 Å x 30 Å was employed for the grid generation.

#### Ligand docking:

The prepared ligand structures and generated grid box was taken for docking and Glide ligand docking module has been used for docking analysis. Glide XP (Extra Precision) docking mode is employed for docking with default parameters setup such as flexible ligand sampling, 10,000 poses of output per docking run for each ligand and writing 1 best pose from 10,000 poses. The top scored ligand-protein complex was further taken for molecular dynamics simulation studies.

### Molecular Dynamics simulation studies:

Molecular dynamic simulation of the docked complex was performed by GROMACS version 2019 [32]. The energy minimization of the docked complex was carried out by employing steepest descent algorithm with 50,000 steps maximum and the GROMOS96 54a7 force field was applied with a 1000 KJmol-1 – nm-1 of energy tolerance. To solvated the complex, SPC (Simple point charge) water model was used in a 1 nm sized cubic box. Necessary Chloride (Cl-) and Sodium (Na+) were added for the neutralization of the system. Periodic boundary conditions were enforced in all directions. LINCS (Linear constraint) [33] algorithm was used for constrain all the bond length inside the system. Long range electrostatics in the system with 0.16 nm Fourier spacing and 1.2 nm cutoff was computed by using Particle mesh Ewald methods (PME) [34].

The regulation of the temperature (310 K) was implemented by using V-rescale weak coupling method. Parrinello-Rahman [35] coupling method was employed for the equilibration of both the NVT and NPT ensembles. In NVT ensemble, constant number of particles (N), volume (V) and temperature (T), a coupling constant of 0.1 ps for 100 ps and a constant temperature of 310K were used. In NPT ensemble, constant number of particles (N), pressure (P) and temperature (T), 1 bar as constant pressure and the same coupling constant parameter were used. This pre-equilibrated system was further taken for 10ns (50,00,000 steps) production molecular dynamics simulation run. The structural coordinates of the system were saved for every 2 ps and analyzed using the analytical tools available in the GROMACS package. The computation was performed using Intel ® Xeon ® CPU 2.00 GHz, Ubuntu a Linux based operating system.

## Results and discussion:

Molecular docking and molecular dynamics simulation studies of laccase-bisphenol-A binding:

The binding affinity of the laccase against different xenobiotics was determined by molecular docking studies in order to find its role in biodegradable efficacy. Docking studies identify the best binding mode of given ligand with its macromolecular target as well as its binding affinity [36]. The list of 2D structures of the corresponding xenobiotic compounds taken for the docking studies is given in supplementary material. Molecular docking scores such as Glide XP GScore, Glide XP energy (kcal/mol), Glide XP Emodel (kcal/mol) of the xenobiotics are reported in (Table 2) (see supplementary material). The best fitted compound Bisphenol is therefore investigated at molecular level and the details of interaction are presented in (Table 1) (see supplementary material).

**Figure 1:**
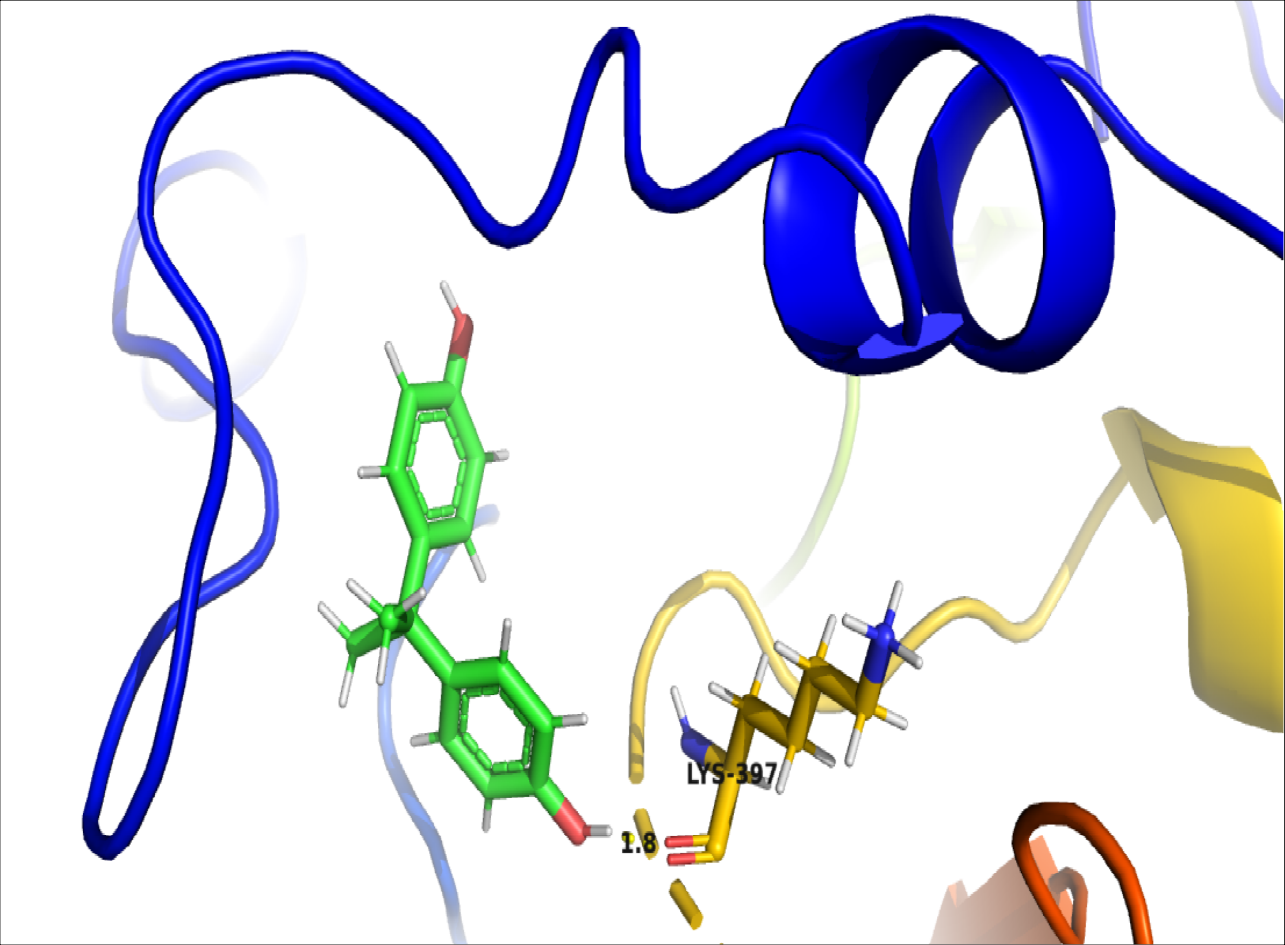
Extra precision (XP) Glide docking depicting the H-Bond interaction between Bisphenol and LYS397 residue of the laccase.

The molecular docking studies have revealed the Glide score of -5.44 and the Glide energy -37.65 kcal/mol for the bisphenol-A bounded at the active site of laccase. The extra precision glide docking interaction reveals the hydrogen bond interaction between -OH group of LYS397 and O1 atom of Bisphenol with the bond length of 1.8 Å. The protein docking simulation indicated the binding of bisphenol-A with the active site of laccase enzyme, which is depicted in cartoon model and bisphenol-A in ball and stick model representation (Figure 1).

**Figure 2:**
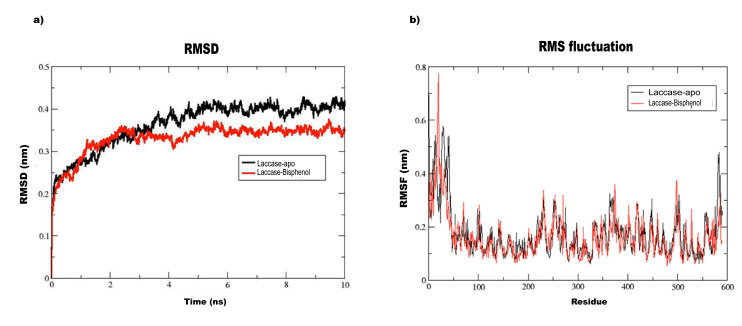
(a) Root mean square deviation (RMSD) plot of Laccase-apo (Black) and Laccase-Bisphenol A (Red) complex at10 ns of molecular dynamic simulation. (b) The RMSF plot of the backbone heavy atoms of the Laccase-apo (Black) and Laccase-Bisphenol A (Red) complex.

The Apo structure of laccase and bisphenol-laccase docked complex were subjected to molecular dynamics simulation studies for 10 ns simulation run. The stability of the apo and docked complexes were analyzed by RMSD, RMSF, radius of gyration, SASA and PCA plots. The RMSD plot shows the bisphenol-laccase docked complex is more stable as compared to the apo structure with lesser deviation (Figure 2a). The root mean square (RMS) fluctuations have provided the structural flexibility of the enzyme in the region of residues (Asn 27 – Leu 32) ranging from 0.3 to 0.7 and suggesting that these residues were involved in the loop formation (Figure 2b). The analysis of both laccase-apo and bisphenol-laccase docked complex by RMSD and RMSF plots have clearly depicted that bisphenol-laccase docked complex is more stable than the laccase-apo complex.

**Figure 3:**
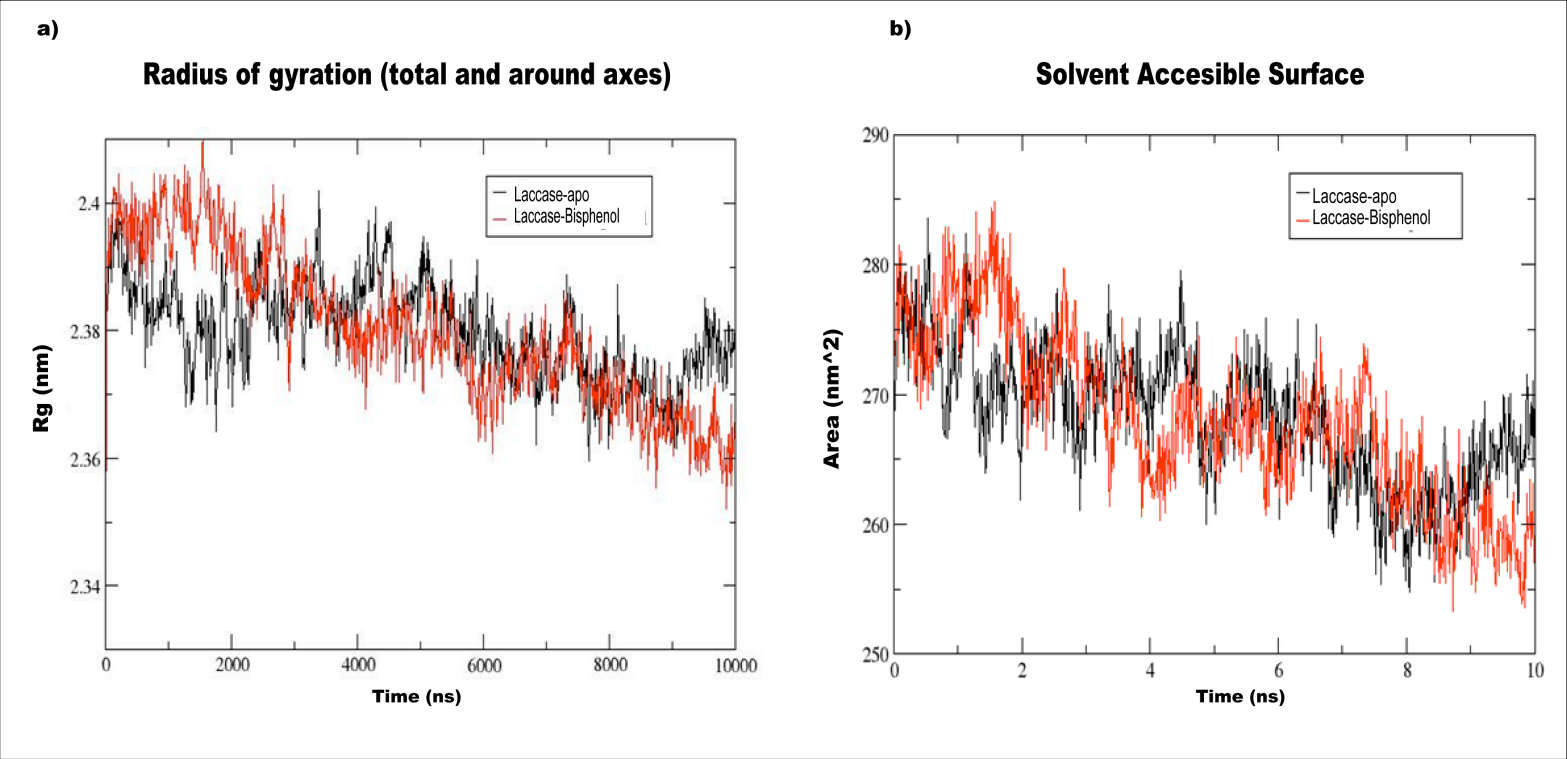
(a) Radius of gyration (Rg) plots of laccase-apo (Black) and laccase-Bisphenol A (Red) complex over the simulation time. (b) SASA plots of Laccase-apo (Black) and Laccase-Bisphenol A (Red) docked complexes from 0–10ns time scale.

The compactness of the protein structure is measured by the radius of gyration (Rg) and the Rg of apo-laccase and bisphenol-laccase docked complex was in the range of 2.38 nm. The relatively steady Rg value suggests that the protein is folded stably and the radius of gyration plot is shown in (Figure 3a). From the figure, the Rg value of 2.4 nm was obtained for bisphenol-laccase and 2.39 nm for laccase-apo at 2000 ps and almost remained the same for the rest of the simulation. Even at 10000 ps, the Rg value between the laccase-apo and bisphenol-laccase was almost similar (2.37-2.38 nm). The results further indicated that the Trichoderma laccase could maintain the structural stability even after binding with the bisphenol-A. Backbone RMSD calculations were carried out for the Trametes versicolor laccase and lignin model compounds (2,6-dimethoxyphenol, ferulic acid, guaiacol, sinapic acid and vanillyl alcohol) at every 10 ps and it was reported that the laccase-lignin model complexes were quickly reach the equilibrium, with the maximum deviation of < 2.9 Å [37]. It was further stated that the Rg of any analyzed complex did not fluctuate sharply during whole simulation except for the initial stage thus represents the stability of the enzyme-substrate complex.

The solvent accessible surface area or SASA were measured on both the bound (Lac-BPA) and unbound (the free enzyme) and the difference between the complexes was presented in (Figure 3b). The SASA of the Bisphenol-laccase docked complex has shown less deviation when compared to the apo-laccase enzyme. The solvent accessible surface area of apo and docked complexes were ranging from 255-280 nm. The SASA values of the docked complex are slightly lower than the apo structure at the end of 10 ns simulation time. From the SASA plot it is evident that the bisphenol-laccase docked complex is having constant accessibility when compared to apo-laccase enzyme. It was reported that the overall folding of amino acids of Lac in LP1 (Lac and phenol) and LP2 (Lac, phenol and Triton X-100) was accompanied by a reduction of its solvent accessible surface area (SASA) and it exhibited the largest SASA value in its stretched conformation [38].

**Figure 4:**
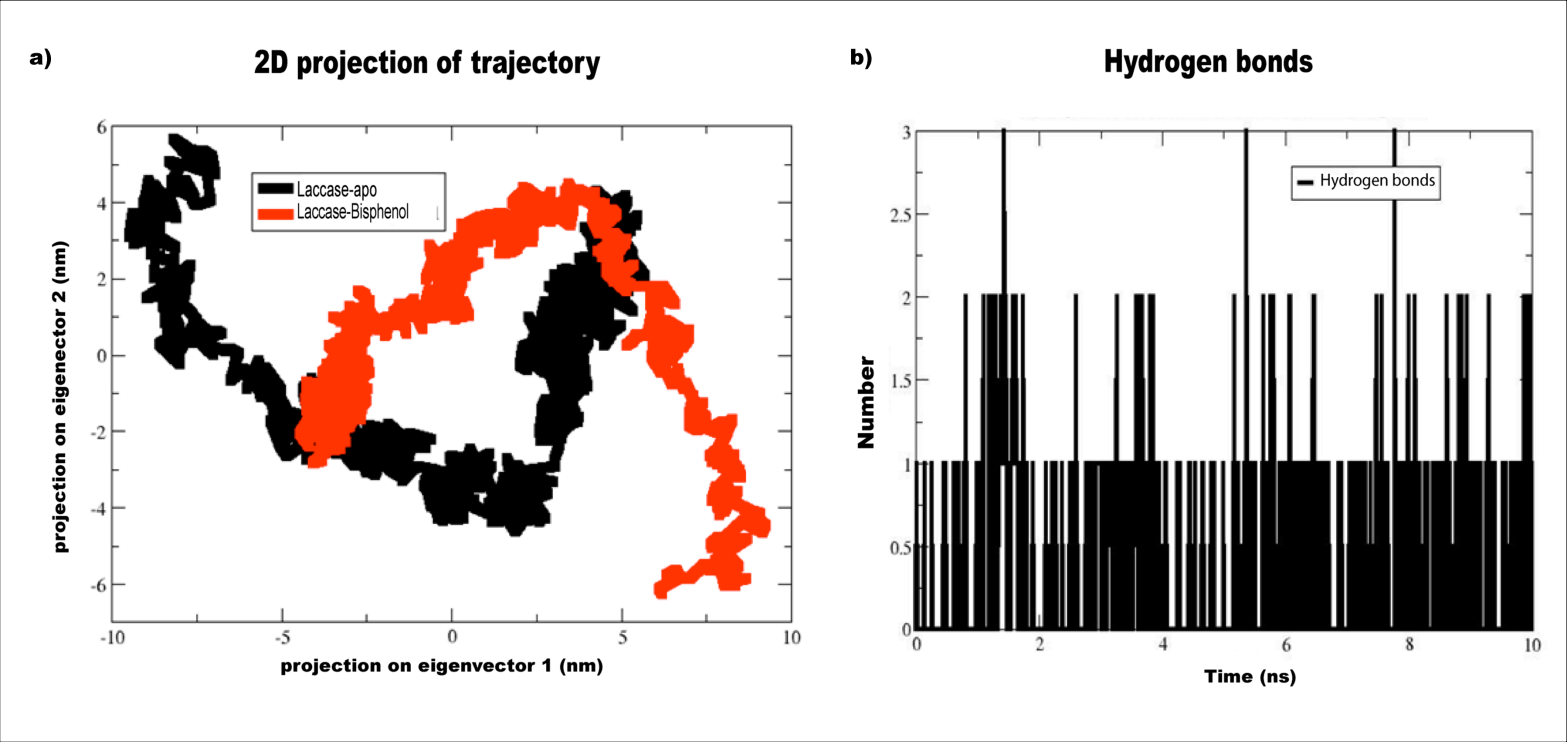
(a) PCA plots of laccase-apo (Black) and laccase-Bisphenol A (Red) complex. (b) Total H-bond interaction of Bisphenol with the residues of laccase enzyme over 10 ns simulation time.

The principal component analysis (PCA) was performed to identify the global motions of the lac-protein in apo-state and laccase-bisphenol complex and the results were shown in (Figure 4a). The comparison of laccase-apo (Black) and laccase-Bisphenol (Red) exhibits lesser motion in laccase-bisphenol compared to apo-laccase. Hence, the results revealed that the laccase-bisphenol docked complex is highly stable and it was projected that the binding of the bisphenol-A stabilizes the laccase protein. The PCA will normally use the trajectory of a molecular dynamics simulation and extracts the dominant modes in the motion of the molecule. Further, these pronounced motions correspond to correlate vibrational modes or collective motions of groups of atoms in normal mode analysis [39].

Studying the hydrogen bond interactions is crucial in different chemical and biological processes such as ligand binding and enzyme catalysis. Hence, bisphenol-A binding to the laccase catalytic site was analyzed by 10 ns MD simulation trajectories to understand the nature of H-bonding interactions. The number of hydrogen bonds with a function of time in the laccase catalytic site contains bisphenol-A shows the one hydrogen bonding interaction was found constantly throughout the simulation period (Figure 4b). Additionally, 2 and 3 hydrogen bonding interaction was observed with lesser extent at 10 ns MD simulation. The hydrogen bonding interaction simulation plot indicated a strong affinity between laccase and bisphenol-A via one H-Bond interaction. Hydrogen bonding interactions are the most stable in biological macromolecules because of their flexible and ubiquitous nature and these interactions will influence the binding specificity, ADME (adsorption, distribution, metabolism and excretion) properties of small molecules.

## Conclusions:

Fungal laccases are widely known for the degradation of recalcitrant xenobiotic compounds. Hence, it is of interest to study the interaction between laccase from Trichoderma laccase and endocrine-disrupting chemical (EDC) named Bisphenol A. The molecular docking analysis of laccase from Trichoderma laccase with 23 xenobiotics and Bisphenol A was completed. We show Bisphenol having optimal binding features (Glide score of -5.44 and the Glide energy -37.65 kcal/mol) with laccase from Trichoderma laccase in this report.
